# Luteolin Inhibits Vascular Smooth Muscle Cell Proliferation and Migration by Inhibiting TGFBR1 Signaling

**DOI:** 10.3389/fphar.2018.01059

**Published:** 2018-09-21

**Authors:** Yu-Ting Wu, Ling Chen, Zhang-Bin Tan, Hui-Jie Fan, Ling-Peng Xie, Wen-Tong Zhang, Hong-Mei Chen, Jun Li, Bin Liu, Ying-Chun Zhou

**Affiliations:** ^1^School of Traditional Chinese Medicine, Southern Medical University, Guangzhou, China; ^2^Department of Traditional Chinese Medicine, Nanfang Hospital, Southern Medical University, Guangzhou, China; ^3^Guangzhou Institute of Cardiovascular Disease, The Second Affiliated Hospital of Guangzhou Medical University, Guangzhou, China

**Keywords:** luteolin, vascular smooth muscle cell, proliferation, migration, TGFBR1, Smad2/3

## Abstract

Vascular smooth muscle cell (VSMC) proliferation and migration play a critical role in the development of arterial remodeling during various vascular diseases including atherosclerosis, hypertension, and related diseases. Luteolin is a food-derived flavonoid that exerts protective effects on cardiovascular diseases. Here, we investigated whether transforming growth factor-β receptor 1 (TGFBR1) signaling underlies the inhibitory effects of luteolin on VSMC proliferation and migration. We found that luteolin reduced the proliferation and migration of VSMCs, specifically A7r5 and HASMC cells, in a dose-dependent manner, based on MTS and EdU, and Transwell and wound healing assays, respectively. We also demonstrated that it inhibited the expression of proliferation-related proteins including PCNA and Cyclin D1, as well as the migration-related proteins MMP2 and MMP9, in a dose-dependent manner by western blotting. In addition, luteolin dose-dependently inhibited the phosphorylation of TGFBR1, Smad2, and Smad3. Notably, adenovirus-mediated overexpression of TGFBR1 enhanced TGFBR1, Smad2, and Smad3 activation in VSMCs and partially blocked the inhibitory effect of luteolin on TGFBR1, Smad2, and Smad3. Moreover, overexpression of TGFBR1 rescued the inhibitory effects of luteolin on the proliferation and migration of VSMCs. Additionally, molecular docking showed that this compound could dock onto an agonist binding site of TGFBR1, and that the binding energy between luteolin and TGFBR1 was -10.194 kcal/mol. Simulations of molecular dynamics showed that TGFBR1-luteolin binding was stable. Collectively, these data demonstrated that luteolin might inhibit VSMC proliferation and migration by suppressing TGFBR1 signaling.

## Introduction

Atherosclerosis is a chronic inflammatory disease of the arteries and is the leading cause of morbidity and mortality worldwide (Weber and von Hundelshausen, 2017). VSMCs are one of the principal components of the arterial walls. Recent evidence indicates that abnormal VSMC proliferation and migration play a vital role in the pathologies and development of AS (Lacolley et al., 2012). Therefore, suppressing the proliferation and migration of these cells could play an important role in preventing the pathological process of AS and might become a novel therapeutic strategy.

Luteolin is a common flavonoid that is part of a group of potentially chemoprotective compounds that widely exist in many Huoxue Huatan Chinese herbal medicines (Radix Salviae, Carthami Flos, *Spatholobus suberectus* Dunn, *Platycodon grandiflorus*, Inulae Flos, Asteris Radix Et Rhizoma, etc.), vegetables (carrot, cabbage, artichoke, and celery), fruits (apple), and plant-based beverages (tea) (Wang et al., 2009; Luo et al., 2017). Preclinical *in vitro* and *in vivo* studies have provided reasonable evidence of the cardioprotective effects of luteolin (Lopez-Lazaro, 2009). For example, experimental studies have found that it has multiple pharmacological effects including anti-inflammatory, anti-oxidant, anti-proliferative, anti-migrative, anti-apoptotic, and other pharmacological activities, most of which participate in the pathological process of AS and mediate CVD protection (Jia et al., 2015; Nile et al., 2018). Moreover, previous studies have demonstrated that luteolin treatment inhibits the proliferation of VSMCs (Lang et al., 2012; Jiang et al., 2013; Xu et al., 2015). However, the underlying mechanisms associated with its inhibitory effects on VSMC proliferation and migration are still unclear.

TGFBR1, a specific TGF-β receptor, mediates the physiological and pathological function of TGF-β, which was demonstrated to be a major component of the atherosclerotic process (Serralheiro et al., 2017). TGFBR1 is essential for mediating TGF-β-induced effects on nearly all cell types of the cardiovascular system, including endothelial cells, VSMCs, myofibroblasts, and macrophages (Goumans and Ten Dijke, 2018). It was reported that a TGFBR1 inhibitor suppressed intimal hyperplasia in a mouse model of transplant arteriosclerosis and reduced VSMC proliferation *in vitro* (Sun et al., 2014). Thus, targeting TGFBR1 might represent a strategy for AS treatment. However, it remains to be determined whether luteolin prevents VSMC proliferation and migration through TGFBR1 inhibition. In this study, an *in vitro* model of VSMC proliferation and migration was utilized to investigate if TGFBR1 signaling is involved in the inhibitory effects of luteolin on VSMC proliferation and migration.

## Materials and Methods

### Materials

Antibodies targeting TGFBR1, proliferative cell nuclear antigen (PCNA), Cyclin D1, MMP2, and MMP9 were purchased from Abcam (Cambridge, MA, United States). The p-TGFBR1 (Ser 165) antibody was purchased from MyBioSource (San Diego, CA, United States). Antibodies specific for Smad2, Smad3, p-Smad2 (Ser 465/Ser 467), p-Smad3 (Ser 423/Ser 425), BAX, BCL-2, and GAPDH were purchased from Cell Signaling Technology (Beverly, MA, United States). Crystal Violet Staining Solution was purchased from KeyGEN BioTECH (Nanjing, China). Luteolin was purchased from Chengdu Mansite Biotechnology (Chengdu, China). Other reagents used in this study were obtained from reagent companies.

### A7r5 and HASMC Cell Culture

A7r5 and HASMC cell lines were purchased from the Institute of Biochemistry and Cell Biology Cell Bank (Shanghai Institutes for Biological Sciences, Chinese Academy of Sciences, Shanghai, China). A7r5 and HASMC cells were incubated in DMEM (Gibco, Grand Island, NY, United States) supplemented with 10% fetal bovine serum (Gibco) at 37°C in an atmosphere of 5% CO_2_ and 95% air.

### Adenovirus Preparation

To verify the effect of luteolin, we used recombinant adenovirus, including those used for overexpression of TGFBR1 and control vectors expressing a GFP marker (Ad-GFP), which were purchased from Vigene Biosciences (Shandong, China). A7r5 and HASMC cells were infected with adenovirus for 4 h, at a multiplicity of infection (MOI) of 100, with serum-free DMEM. Medium was replaced with DMEM containing 10% fetal bovine serum for 48 h before performing different treatments.

### Cell Proliferation Assay

The Cell Titer 96^®^ Aqueous One Solution Cell Proliferation Assay (MTS, Promega, Madison, WI, United States) was used to detect the effect of luteolin on the proliferation of A7r5 and HASMC cells according to the manufacturer’s protocol. Briefly, A7r5 and HASMC cells in logarithmic growth phase were harvested and seeded in 96-well plates at a density of 5 × 10^3^ cells/well. Upon adherence to the plates, cells were starved with serum-free DMEM overnight. Next, they were treated with different concentrations of luteolin (10, 20, and 40 μM) and incubated for 48 h. MTS solution was directly added to each well (20 μl/well), and cells were cultured for 1 h; the optical density (OD) at 490 nm was measured for each well using an automatic microplate reader (Gene Company, Hong Kong, China).

### 5-Ethynyl-2′-Deoxyuridine Proliferation Assay

EdU was used to detect the effect of luteolin on the proliferation of A7r5 and HASMC cells according to the Cell-Light^TM^ EdU Apollo^®^488 *In Vitro* Imaging Kit (Guangzhou RiboBio, China) instructions. Briefly, cells were treated with MTS as described in Section “Cell Proliferation Assay.” Then, EdU (100 mM, 100 μl/well) was added to the 96-well plates, which were incubated for 12 h. Next, A7r5 and HASMC cells were fixed with 4% paraformaldehyde. After 30 min, 1× Apollo^®^ staining reaction liquid was added (100 μl/well), and cells were incubated for 30 min at room temperature; after 10 min of permeabilization with 0.5% Triton X-100, the cells were stained with 1× Hoechst 33342 (100 μl/well) for 30 min. The ratio of EdU-positive cells (EdU-stained cells/Hoechst-stained cells × 100%) was determined using a fluorescence microscope (Nikon Eclipse Ti-S, Tokyo, Japan).

### Transwell Migration Assay

A Transwell chamber was used to detect the migrative ability of A7r5 and HASMC cells. Briefly, A7r5 and HASMC cells were seeded into the upper chamber at a density of 1 × 10^5^ cells/well and treated with different concentrations of luteolin (10, 20, and 40 μM) with serum-free DMEM. The lower chambers, which were devoid of cells, were filled with DMEM with 10% fetal bovine serum and placed in 24-well plates. After incubation for 12 h, cells on the upper surface had migrated through the micropores (8-μM pores), and Transwell chambers were washed with phosphate-buffered saline three times; the lower side was fixed with 4% paraformaldehyde. After 20 min, the migrated cells were stained with crystal violet staining solution for 15 min. Five visual fields were randomly selected from each Transwell filter and captured at 400× magnification using an inverted fluorescence microscope (Nikon Eclipse Ti-S). The average number of cells that migrated through the Transwell filter was assayed using ImageJ software.

### Wound Healing Migration Assay

Wound healing assays were used to detect the migrative ability of A7r5 and HASMC cells. Briefly, A7r5 and HASMC cells were treated with different concentrations of luteolin (10, 20, and 40 μM) for 24 h in DMEM containing 1% fetal bovine serum. Then, monolayers were wounded with a sterile 10-μl pipette tip, and suspended cells were washed away with phosphate-buffered saline twice. Images were captured of each well at 200× magnification at 0 and 48 h. Migration distance was estimated based on the widths of the wounds at 0 and 48 h, which were assayed using ImageJ software.

### Western Blot Analysis

In brief, protein extracts from cultured cells were prepared in RIPA buffer containing protease and phosphatase inhibitors (all purchased from Beyotime, Shanghai, China). Lysates were centrifuged at 12,000 rpm at 4°C for 15 min to obtain the supernatant. Approximately 15 μg of protein was separated on a 10% SDS-PAGE gel and transferred to a polyvinylidene difluoride membrane (Millipore, Bedford, MA, United States), which was blocked with Tris-buffered saline containing 5% non-fat milk. Target proteins were incubated with the corresponding primary antibodies overnight at 4°C as follows: p-TGFBR1, 1:1,000; total TGFBR1, 1:1,000; p-Smad2, 1:1,000; p-Smad3, 1:1,000; total Smad2, 1:1,000; total Smad3, 1:1,000; Cyclin D1, 1:10,000; PCNA, 1:1,000; MMP2, 1:1,000; MMP9, 1:1,000; BAX, 1:1,000; BCL-2, 1:1,000; GAPDH, 1:1,000. Membranes were then washed three times with Tris-buffered saline containing 0.1% Tween 20 (TBST) and incubated with horseradish peroxidase-goat anti-rabbit IgG (Beverly, MA, United States) for 2 h at 4°C. GAPDH was used as an internal control. Band intensity was quantified using ImageJ software.

### Molecular Docking and Simulation of Molecular Dynamics

Molecular docking, which was analyzed by Autodock Vina (Scripps Research Institute, United States) (Dos Santos et al., 2018), was used to analyze the binding mechanism between TGFBR1 (PDB ID: 1PY5) and luteolin (ZINC ID: 5280445). The molecular docking simulation protein of TGFBR1 was prepared by removing water molecules and bound ligands. YASARA was used to perform the energy minimization of ligands. In this study, the agonist-binding domain of TGFBR1 corresponded to the protein-ligand binding site of TGFBR1. The star conformation for the MD simulation was considered the best conformation.

YASARA was used to perform MD simulation (Land and Humble, 2018). AMBER 03 forcefield was used to run all simulations. Briefly, 0.9% NaCl, as the solvation of the receptor-ligand complex, was placed in a dodecahedron box; the distance between the box and the solute as 5 Å. The initiation of simulated annealing minimizations was set at 298 K, with velocities scaling down 0.9 every 10 steps lasting for 5 ps. After the energy was minimized, the temperature of the system was adjusted using a Berendsen thermostat to minimize the influence of temperature control. In addition, velocities were rescaled only every 100 simulation steps, whenever the mean of the last 100 detected temperatures converged. Finally, 100-ns MD simulations were conducted at a rate of 2 fs, and the coordinates of the complexes were saved every 10 ps.

### Data Analysis

All data are expressed as the mean ± SD and were analyzed using the SPSS statistical package (version 13.0, SPSS Inc., Chicago, IL, United States). One-way analysis of variance factorial analysis was used to compare measurement data among the groups. If the variances were homogenous, the mean values were compared by performing an *F*-test. The differences between two groups were analyzed using a Bonferroni test. If the variances were not homogenous, Welch’s test and Dunnett’s T3 analysis were adopted. *P* < 0.05 indicated statistically significant differences. Each experiment was repeated three or more times.

## Results

### Luteolin Inhibits VSMC Proliferation Without Inducing Apoptosis

**FIGURE 1:**
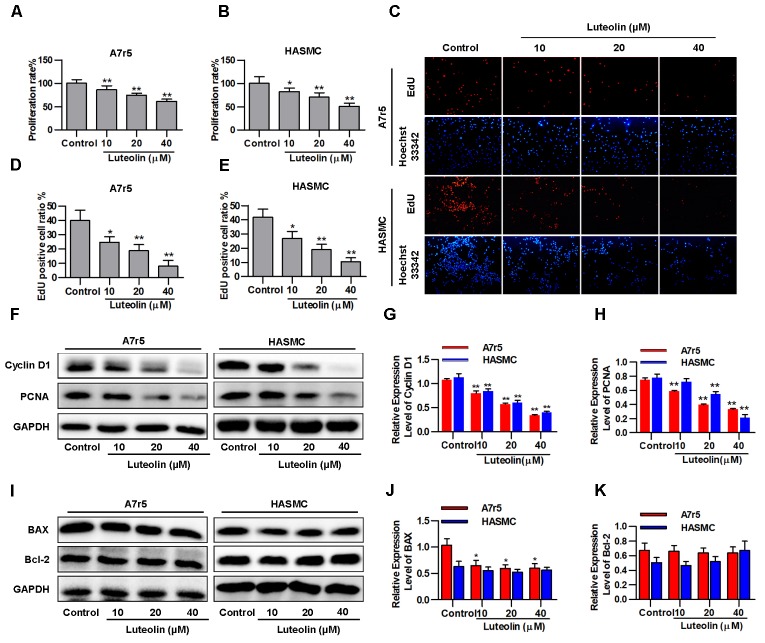
Luteolin suppresses vascular smooth muscle cell (VSMC) proliferation without inducing apoptosis. **(A**,**B)** A7r5 and HASMC cells were incubated with different concentrations of luteolin (10, 20, and 40 μM) for 48 h, and then VSMC proliferation was tested by MTS assays (*n* = 6). **(C)** EdU proliferation assay. The red regions (EdU) in the images represent proliferating cells, and the blue regions (Hoechst 33324) represent the nuclei of all cells. **(D**,**E)** Percentage of EdU-positive VSMCs (the proliferation ratio was tested by assessing Hoechst 33324/EdU staining; *n* = 3). **(F)** A7r5 and HASMC cells were incubated with luteolin (10, 20, and 40 μM) for 24 h, and the expression of Cyclin D1 and PCNA was tested by western blotting. **(G**–**H)** Relative expression levels of Cyclin D1 and PCNA (*n* = 3). **(I)** Expression levels of BAX and BCL-2 were tested by western blotting. **(J)** Relative expression levels of BAX (*n* = 3). **(K)** Relative expression levels of BCL-2 (*n* = 3). Data are presented as the mean ± SD. ^∗^*P* < 0.05, ^∗^*P* < 0.01, compared to the control group.

To detect the effect of luteolin on VSMC proliferation, A7r5 and HASMC cells were treated as described in the experimental section. Then, MTS assays and EdU staining were used to detect the proliferation of VSMCs. As shown in **Figures 1A,B**, treatment with different concentrations of luteolin (10, 20, and 40 μM) significantly inhibited A7r5 and HASMC cell proliferation in a dose-dependent manner (*P* < 0.05). Consistently, the ratios of EdU-positive cell numbers for A7r5 and HASMC cells were also dose-dependently reduced by luteolin (*P* < 0.05, **Figures 1C–E**). In addition, we further detected the inhibitory effect of luteolin through the expression of proliferation-related proteins including Cyclin D1 and PCNA. CyclinD1 is a regulatory protein of the cell cycle and plays a key role in progression from the G1 to S phase in the mammalian cell. PCNA is a well-defined regulator of DNA replication and cell cycle control and is used as a proliferation index of a broader spectrum of cells (Zhao et al., 2013; Kim et al., 2014; Xu et al., 2015). As shown in **Figures 1F–H**, this compound significantly inhibited the expression of Cyclin D1 and PCNA in a dose-dependent manner (*P* < 0.05). Furthermore, to determine if luteolin inhibits cell proliferation by inducing apoptosis, we detected the expression of apoptosis-related proteins by western blotting. As shown in **Figures 1I–K**, we found that luteolin could not induce VSMC apoptosis. These results indicated that luteolin inhibited VSMC proliferation without inducing apoptosis.

### Luteolin Inhibits VSMC Migration

**FIGURE 2:**
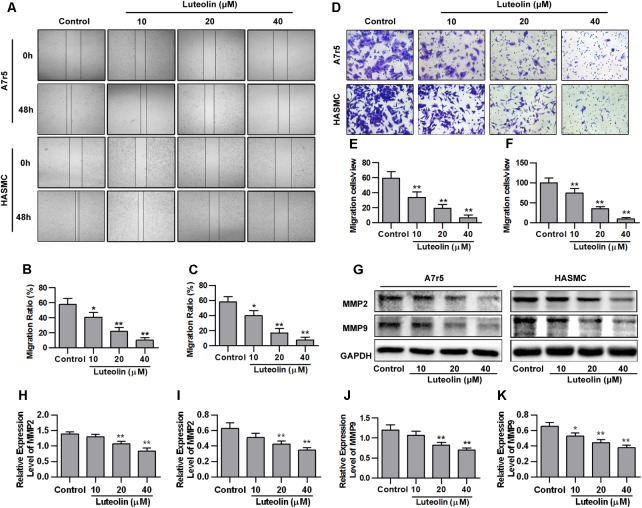
Luteolin suppresses the migration of vascular smooth muscle cells (VSMCs). **(A)** A7r5 and HASMC cells were incubated with different concentrations of luteolin (10, 20, and 40 μM) for 24 h, and the migration of VSMCs was tested by wound healing assays; the migration distance was estimated based on the width of the scrape at 0 and 48 h. **(B**,**C)** Percentage of relative VSMC migration (*n* = 3). **(D)** A7r5 and HASMC cells were harvested in the logarithmic growth phase, treated with different concentrations of luteolin (10, 20, and 40 μM), and tested by performing Transwell assays for 12 h. **(E**,**F)** The number of cells in each field of view (*n* = 5). **(G)** A7r5 and HASMC cells, respectively, were incubated with luteolin (10, 20, and 40 μM) for 24 h, and the expression levels of MMP2 and MMP9 were tested by western blotting. **(H**–**K)** Relative expression levels of MMP2 and MMP9 (*n* = 3). Data are presented as the mean ± SD. ^∗^*P* < 0.05, ^∗∗^*P* < 0.01, compared to the control group.

To detect the effect of luteolin on VSMC migration, wound healing and Transwell assays were performed using A7r5 and HASMC cells. As shown in **Figures 2A–C**, luteolin inhibited A7r5 and HASMC cell migration in a dose-dependent manner (*P* < 0.05). Consistently, as shown in **Figures 2D–F**, the migrative ability of A7r5 and HASMC cells was also dose-dependently reduced by luteolin (*P* < 0.05). In addition, we further detected the expression levels of migration-related proteins (MMP2 and MMP9) to verify these effects. MMPs are a large family of calcium-dependent zinc-containing endopeptidases, which are responsible for tissue remodeling and degradation of the extracellular matrix (ECM). Previous studies have demonstrated that MMP2 and MMP9 are most closely involved in VSMC migration in both *in vitro* and *in vivo* models (Kenagy et al., 1997; Ding et al., 2011; Guo et al., 2013). Selective gene silencing of MMP2 and MMP9, or interruption of MMP2 and MMP9 gene expression by chemical inhibitors, inhibit the migration of VSMCs (Pinney et al., 2003; Hlawaty et al., 2007; Turner et al., 2007). As shown in **Figures 2G–K**, this compound inhibited the expression of MMP2 and MMP9 in a dose-dependent manner (*P* < 0.05). These findings indicated that luteolin inhibits VSMC migration.

### Luteolin Inhibits Activation of the TGFBR1 Signaling Pathway

**FIGURE 3:**
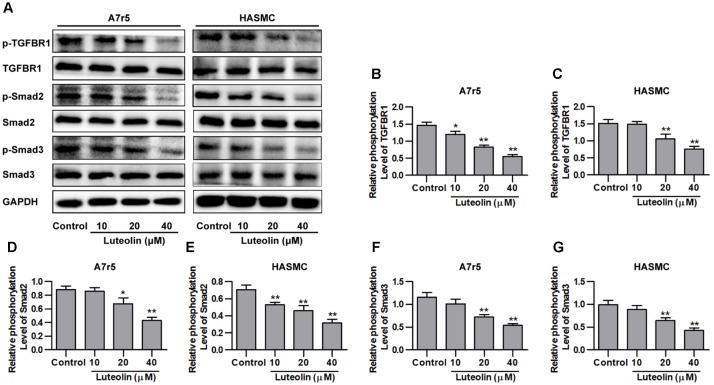
Luteolin suppresses activation of the TGFBR1 signaling pathway. **(A)** A7r5 and HASMC cells, respectively, were incubated with luteolin (10, 20, and 40 μM) for 1 h, and the expression levels of p-TGFBR1, TGFBR1, p-Smad2, Smad2, p-Smad3, and Smad3 were tested by western blotting. **(B**–**G)** Relative phosphorylation levels of TGFBR1, Smad2, and Smad3 (*n* = 3). Data are presented as the mean ± SD. ^∗^*P* < 0.05, ^∗∗^*P* < 0.01, compared to the control group.

Here, we investigated whether luteolin inhibits TGFBR1 signaling. As shown in **Figures 3A–C**, administration of luteolin significantly inhibited the phosphorylation of TGFBR1 in a dose-dependent manner without altering total levels of TGFBR1 (*P* < 0.05). Smad2 and Smad3 are the downstream signaling molecules of TGFBR1, and as shown in **Figures 3A,D–G**, the phosphorylation levels of Smad2 and Smad3 were also reduced with treatment in a dose-dependent manner without altering total levels of Smad2 and Smad3 (*P* < 0.05). In addition, we further evaluated the effects of luteolin on TGF-β-induced TGFBR1 and Smad activation in VSMCs. As shown in **Supplementary Figures S1A,B**, the phosphorylation levels of TGFBR1 were significantly enhanced under TGF-β treatment (*P* < 0.05), suggesting that the binding of TGF-β activates TGFBR1. Consistently, as shown in **Supplementary Figures S1A,C,D**, the phosphorylation levels of Smad2 and Smad3 were also significantly enhanced in TGF-β-stimulated VSMCs (*P* < 0.05). Furthermore, luteolin (40 μM) significantly reduced the phosphorylation levels of TGFBR1, Smad2, and Smad3 (*P* < 0.05). These results strongly indicate that luteolin inhibited the activation of TGFBR1/Smad signaling.

### Overexpression of TGFBR1 Partially Abolishes the Inhibitory Effect of Luteolin on Smads

**FIGURE 4:**
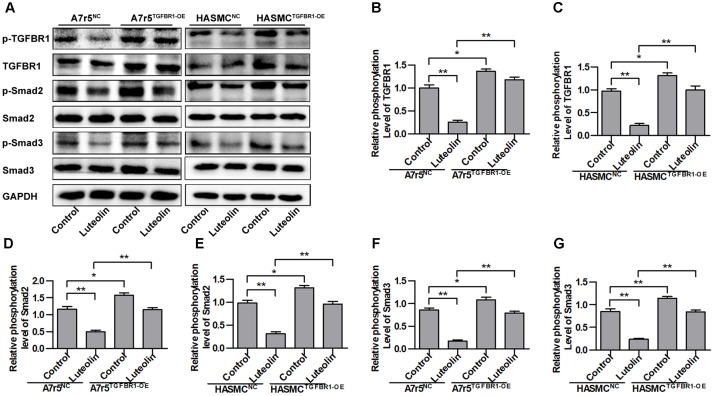
Overexpression of TGFBR1 partially rescues TGFBR1 activation after luteolin treatment. **(A)** A7r5 and HASMC cells, respectively, were incubated with a TGFBR1 overexpression adenovirus vector and GFP control adenovirus for 48 h. Cells were then treated with luteolin (40 μM) for 1 h. The expression levels of p-TGFBR1, TGFBR1, p-Smad2, Smad2, p-Smad3, and Smad3 were tested by western blotting. **(B**–**G)** Relative phosphorylation levels of TGFBR1, Smad2, and Smad3 (*n* = 3). Data are presented as the mean ± SD. ^∗^*P* < 0.05, ^∗∗^*P* < 0.01, compared to the control group.

To determine if the inhibitory effect of luteolin on Smad signaling was due to suppression of TGFBR1, we overexpressed human TGFBR1 in A7r5 and HASMC cells using a recombinant adenovirus (Ad-TGFBR1). After Ad-TGFBR1 and negative control adenovirus (Ad-NC) treatment was administered for 48 h, A7r5 and HASMC cells were treated with luteolin (40 μM). As shown in **Figures 4A–C**, the phosphorylation levels of TGFBR1 were significantly enhanced in TGFBR1-overexpressing VSMCs (*P* < 0.05). The phosphorylation levels of Smad2 and Smad3, which are downstream molecules of TGFBR1, were also measured by western blotting. As presented in **Figures 4A,D–G**, overexpression of TGFBR1 reduced the inhibitory effects of luteolin on the phosphorylation of Smad2 and Smad3 in A7r5 and HASMC cells (*P* < 0.05). Previous studies have demonstrated that luteolin exerts an inhibitory action on VSMC proliferation through suppressing Akt signaling (Lang et al., 2012; Xu et al., 2015). Thus, in this study, we investigated whether luteolin inhibits Akt in a TGFBR1-dependent manner. Interestingly, we found that the phosphorylation level of Akt was reduced in TGFBR1-overexpressing VSMCs (**Supplementary Figures S2A,B**; *P* < 0.05). Treatment with luteolin (40 μM) inhibited the phosphorylation level of Akt; overexpression of TGFBR1 enhanced the inhibitory effects of luteolin on Akt (**Supplementary Figures S2A,B**; *P* < 0.05). These results indicated that luteolin inhibited Smads trough a TGFBR1-dependent manner.

### Overexpression of TGFBR1 Partially Blocks the Inhibitory Effect of Luteolin on VSMC Proliferation

**FIGURE 5:**
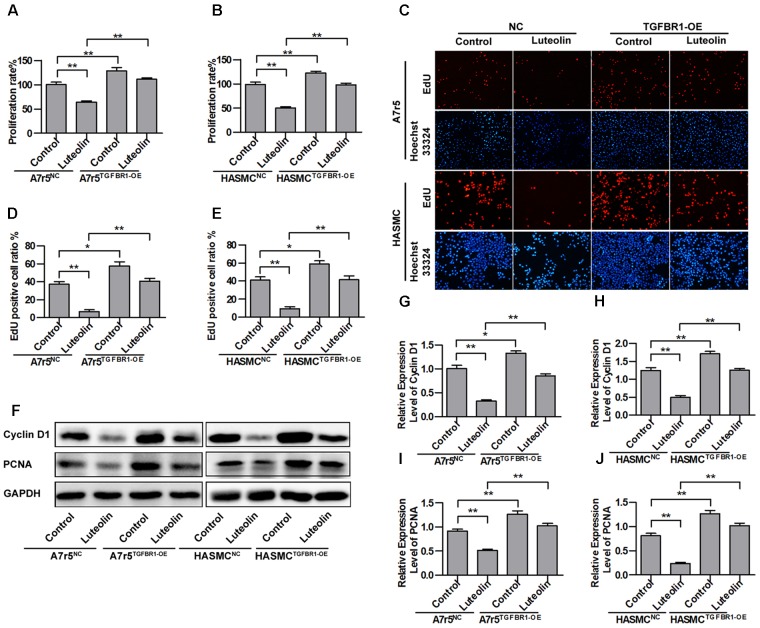
Overexpression of TGFBR1 partially blocks the inhibitory effect of luteolin on vascular smooth muscle cell (VSMC) proliferation. **(A**,**B)** A7r5 and HASMC cells, respectively, were incubated with TGFBR1 overexpression adenovirus vector and GFP control adenovirus for 48 h and then treated with luteolin (40 μM) for 48 h. Then, the proliferation of VSMCs was tested by MTS assays (*n* = 6). **(C)** EdU proliferation assay. The *red* regions (EdU) in the images represent proliferating cells, and the *blue* regions (Hoechst 33324) represent the nuclei of all cells. **(D**,**E)** Percentage of EdU-positive VSMCs (*n* = 3). **(F)** A7r5 and HASMC cells, respectively, were incubated with TGFBR1 overexpression adenovirus vector and GFP control adenovirus for 48 h and then treated with luteolin (40 μM) for 24 h. the expression levels of Cyclin D1 and PCNA were tested by western blotting. **(G**–**J)** Relative expression levels of Cyclin D1 and PCNA (*n* = 3). Data are shown as the mean ± SD. ^∗^*P* < 0.05, ^∗∗^*P* < 0.01.

To further verify that the inhibitory effects of luteolin on VSMC proliferation are associated with inhibiting the activation of TGFBR1, we overexpressed TGFBR1 in A7r5 and HASMC cells and then detected the anti-proliferative effects of luteolin. As shown in **Figures 5A,B**, overexpression of TGFBR1 significantly enhanced the proliferation of luteolin-treated A7r5 and HASMC cells (*P* < 0.05). As shown in **Figures 5C–E**, consistently, overexpression of TGFBR1 also significantly reduced the inhibitory effect of luteolin on the EdU-positive cell ratio in both cell lines (*P* < 0.05). Next, we further detected the expression levels of cell cycle-related proteins including Cyclin D1 and PCNA. As shown in **Figures 5F–J**, the expression of Cyclin D1 and PCNA was partially rescued by overexpression of TGFBR1 (*P* < 0.05). These findings indicated that the inhibitory effects of luteolin on VSMC proliferation are at least partly due to inhibition of TGFBR1 activation.

### Overexpression of TGFBR1 Partially Blocks the Inhibitory Effect of Luteolin on VSMC Migration

**FIGURE 6:**
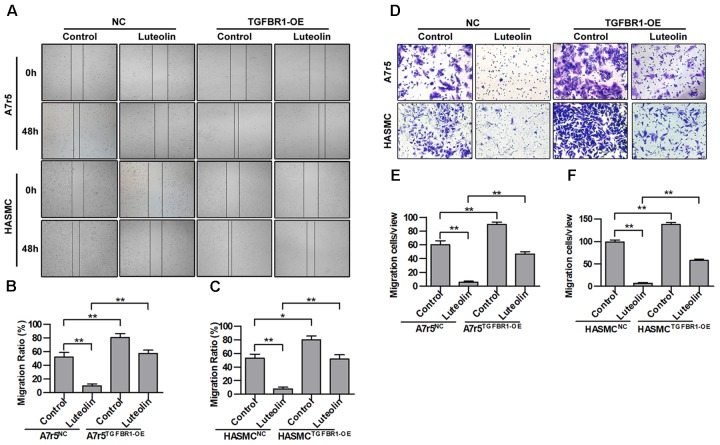
Overexpression of TGFBR1 partially blocks the inhibitory effect of luteolin on vascular smooth muscle cell (VSMC) migration. **(A)** A7r5 and HASMC cells were incubated with TGFBR1 overexpression adenovirus vector and GFP control adenovirus for 48 h and then treated with luteolin (40 μM) for 24 h. Then, the migration of VSMCs was tested by wound healing assays. **(B**,**C)** Percentage of relative VSMC migration (*n* = 3). **(D)** A7r5 and HASMC cells were incubated with TGFBR1 overexpression adenovirus vector and GFP control adenovirus for 48 h. Then, Transwell assays were performed after treatment with luteolin (40 μM) for 12 h. **(E**,**F)** The number of cells in each field of view (*n* = 5). Data are shown as the mean ± SD. ^∗^*P* < 0.05, ^∗∗^*P* < 0.01.

Next, we further determined whether the inhibitory effect of luteolin on VSMC migration was due to inhibited activation of TGFBR1. TGFBR1 was overexpressed in A7r5 and HASMC cells using Ad-TGFBR1, and the anti-migration effects of luteolin were measured by wound healing and Transwell assays. As shown in **Figures 6A–C**, overexpression of TGFBR1 significantly enhanced the migration of luteolin-treated A7r5 and HASMC cells (*P* < 0.05). As shown in **Figures 6D–F**, consistently, overexpression of TGFBR1 also partially blocked the inhibitory effect of luteolin on the number of migrating cells, and this difference was statistically significant (*P* < 0.05). Our findings also suggested that the inhibitory effects of luteolin on VSMC migration were due to, at least in part, inhibition of TGFBR1 activation.

### Luteolin Directly Targets TGFBR1

**FIGURE 7:**
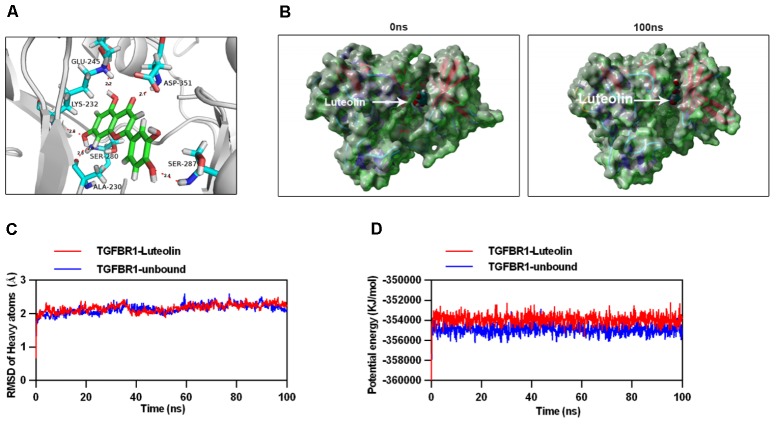
Molecular docking and simulation of the molecular dynamics. **(A)** Three-dimensional crystal structure of luteolin (ZINC18185774) in complex with TGFBR1 (PDB ID: 1PY5). Luteolin is shown in *green*, and the hydrogen bonds are indicated by *sky-blue lines*. **(B)** Surface presentation of the TGFBR1-luteolin complex crystal structure at 0 and 100 ns. **(C)** Plots of root mean square deviation (RMSD) of heavy atoms of TGFBR1 unbound (*blue*) and TGFBR1-luteolin complex (*red*). **(D)** Potential energy profiles of TGFBR1-free (*blue*) and TGFBR1-luteolin complex (*red*) during the 100-ns molecular dynamics simulation.

To explore the interaction between luteolin and TGFBR1, we performed molecular docking analysis, using the docking program Autodock Vina. The binding energy of the TGFBR1-luteolin complex was found to be -10.194 kcal/mol, which indicated good binding ability. The three-dimensional binding conformation of the TGFBR1-luteolin complex is presented in **Figure 7A**. We found six hydrogen bonds that formed between luteolin and GLU-245, LYS-232, ASP-351, SER-280, SER-287, and ALA-230 of TGFBR1. Next, to further verify the results of molecular docking, the best conformation of TGFBR1-luteolin was taken as the start conformation for MD simulation by YASARA. The surface visualization models of the TGFBR1-luteolin complex are shown in **Figure 7B**. Luteolin steadily presented at the center of the TGFBR1 binding site until the end of the MD simulation. In addition, as shown in **Figure 7C**, both the heavy atoms RMSD track of the TGFBR1-luteolin complex (**Figure 7C**, red line) and unbound TGFBR1 (TGFBR1-free) (**Figure 7C**, blue line) mildly fluctuated around 2 Å during 0–100 ns (**Figure 7C**). The track of potential energy of the TGFBR1-luteolin complex was higher than that of unbound TGFBR1 (**Figure 7D**). These results suggested that binding between TGFBR1 and luteolin is stable, and that luteolin might directly target TGFBR1.

## Discussion

Under physiological conditions, VSMCs regulate vascular tone through contraction and relaxation and maintain normal vascular function by secreting and releasing vascular regulatory factors. However, VSMC migration from the media to the subendothelial space and abnormal proliferation represent common pathological bases of AS, hypertension, and vascular restenosis. Luteolin is a common flavonoid that widely exists in many Chinese herbal medicines, vegetables, and fruits. Several studies have demonstrated that luteolin has multiple cardio-protective effects via complex signal transduction pathways and target effectors. In addition, epidemiological evidence has shown that intake of these compounds helps prevent CVD (Luo et al., 2017). These results suggest that generous dietary intake of flavonoids may be an effective primary prevention strategy against CVD. Relevant studies have indicated that luteolin prevents VSMC remodeling, using *in vivo* models. For example, Zhang et al. (2009) reported that flavonoids inhibit neointima formation. Su et al. (2015) reported that luteolin effects decline in media thickness of vascular wall and attenuates hypertensive vascular remodeling through inhibiting the proliferation and migration of VSMCs. In this study, we have demonstrated the inhibitory effect of luteolin on VSMC proliferation and migration. Our study will enrich the current knowledge on the role of luteolin on VSMC proliferation and migration, similar to previous studies (Lang et al., 2012; Jiang et al., 2013; Xu et al., 2015).

TGF-β is a multifunctional cytokine that regulates various cell functions (Agrotis et al., 2005; Weiss and Attisano, 2013) including smooth muscle cell proliferation and migration (Goumans et al., 2009). It has been demonstrated that TGF-β binds TGFBR2 and then induces the phosphorylation of TGFBR1 at Ser165 (Souchelnytskyi et al., 1996). In the present study, we found that luteolin significantly inhibits phosphorylation of TGFBR1 at Ser165 in a dose-dependent manner. Activated TGFBR1 specifically recognizes and phosphorylates Smad2/3, following which phosphorylated Smad2/3 translocates to the nucleus with Smad4 to activate various transcriptional programs through binding to a Smad-binding element (Weiss and Attisano, 2013). Therefore, we further investigated the inhibitory effects of luteolin on the phosphorylation of Smad2/3. We found that treatment with luteolin reduced the phosphorylation levels of Smad2 and Smad3 in a dose-dependent manner. Our findings are in agreement with a previous study showing that luteolin inhibits Smad2 activation in hepatic stellate cells (Li et al., 2015). Then, we further determined whether the inhibitory effects of luteolin on the activation of Smad2/3 were due to TGFBR1 inhibition by overexpressing this receptor. We found that the inhibitory effects of this compound were partly counteracted by TGFBR1 overexpression. These data suggested that luteolin inhibits activation of Smad2/3 via inhibition of TGFBR1.

Next, we further determined whether the inhibitory action of luteolin on VSMC proliferation and migration is associated with its ability to inhibit the activation of TGFBR1 using a TGFBR1-overexpression adenovirus vector. We found that such inhibitory effects were partially blocked with exogenous TGFBR1 expression. These results demonstrated that TGFBR1 is the pharmacological target of luteolin with respect to VSMC proliferation and migration. In addition, the essential involvement of TGFBR1 has been detected in a series of pathophysiological processes associated with CVDs, such as cardiac fibrosis, vascular restenosis, and inflammation (Kajdaniuk et al., 2013; Goumans and Ten Dijke, 2018). Our findings suggest that luteolin could be used to regulate these processes.

The present findings suggested that luteolin might target TGFBR1. Thus, we tested its ability to target TGFBR1 directly by performing molecular docking and MD simulation to assess the mechanism through which luteolin and TGFBR1 interact. The binding energy of the luteolin-TGFBR1 complex indicated strong binding. Moreover, luteolin interacted with GLU-245, LYS-232, ASP-351, SER-280, SER-287, and ALA-230 of TGFBR1 via hydrogen bonds. These residues are the key residues involved in inhibition of the binding domain of TGFBR1 (Sawyer et al., 2004). Furthermore, MD simulation results demonstrated that the luteolin-TGFBR1 binding conformation was stable. These results strongly indicated that luteolin directly targets TGFBR1, acting as a potential TGFBR1 inhibitor.

Multi-targets and complex signaling is an important characteristic of natural products. Previous studies have demonstrated that luteolin inhibits the proliferation and migration of VSMCs by regulating Akt (Lang et al., 2012; Xu et al., 2015). To determine whether luteolin inhibits Akt through suppressing TGFBR1 signaling, we investigated the effect of luteolin on the phosphorylation level of Akt both in TGFBR1-overexpressing and negative control VSMCs. Interestingly, we found the phosphorylation level of Akt to be reduced in TGFBR1-overexpressing VSMCs. Previous studies have shown that treatment with TGF-β increased Akt activation in type 2 diabetic nephropathy mice model and cultured human podocytes (Madne and Dockrell, 2018; Zhang et al., 2018). Different roles of TGFBR1 in Akt signaling might be cell-type-dependent. We also found that luteolin inhibited the phosphorylation level of Akt; overexpression of TGFBR1 significantly enhanced the inhibitory effect of luteolin on Akt, while the activation of Smad2 and Smad3 was increased and the proliferation of VSMC was further enhanced. These results strongly indicate that luteolin performs its inhibitory function on Akt in a TGFBR1-independent manner. Inhibition of TGFBR1 signaling plays a more important role in the inhibitory actions of luteolin than suppression of Akt.

In this study, we demonstrated that luteolin inhibits VSMC proliferation and migration by suppressing TGFBR1 signaling without inducing cell injury. Our findings provide novel insights into the anti-atherosclerotic mechanisms of luteolin. However, there are some limitations to our study. Firstly, we investigated this new potential mechanism of luteolin only *in vitro*, and did not further validate it in animal studies. Secondly, we did not investigate the effective therapeutic dosage and the safety of long-term administration of luteolin. Our future studies will further investigate the molecular mechanisms of luteolin both *in vivo* and *in vitro*.

In summary, a series of experiments was designed to clarify whether TGFBR1 signaling underlies the inhibitory effects of luteolin on VSMC proliferation and migration. The main findings in the present study are as follows. (i) Luteolin inhibited the proliferation and migration of A7r5 and HASMC cells, without affecting apoptosis. (ii) Luteolin decreased TGFBR1 and Smad2/3 phosphorylation. (iii) Overexpression of TGFBR1 relieved the inhibitory effects of luteolin on VSMC proliferation and migration, and phosphorylation of TGFBR1 pathway-associated proteins. (iv) Molecular simulation demonstrated strong and stable binding between luteolin and TGFBR1.

## Author Contributions

Y-TW supervised the entire work and performed the cell cultures and proliferation and migration assays. LC and Z-BT performed the cell cultures and adenovirus transfection. L-PX and W-TZ performed the molecular docking and simulation of molecular dynamics. H-MC and JL analyzed the data. Y-CZ and BL conceived and designed the experiments and critically revised the manuscript. All authors discussed the results and contributed to manuscript writing.

## Conflict of Interest Statement

The authors declare that the research was conducted in the absence of any commercial or financial relationships that could be construed as a potential conflict of interest.

## Abbreviations

AS: atherosclerosis
CVD: cardiovascular diseases
DMEM: Dulbecco’s modified Eagle’s medium
Edu: 5-ethynyl-2′-deoxyuridine
GFP: green fluorescent protein
TGFBR1: transforming growth factor beta receptor 1
VSMC: vascular smooth muscle cell
