# Supplementary material for: Luteolin Inhibits Vascular Smooth Muscle Cell Proliferation and Migration by Inhibiting TGFBR1 Signaling
Source: Front Pharmacol. 2018 Sep 21;9:1059. doi: 10.3389/fphar.2018.01059 (PMC6160560; doi:10.3389/fphar.2018.01059)
Supplement: Supplementary file 1 [file Table_1.DOCX]

Supplementary Material

Luteolin Inhibits Vascular Smooth Muscle Cell Proliferation and[Migration](https://www.ncbi.nlm.nih.gov/pubmed/29324316)by Inhibiting TGFBR1 Signaling

Yu-ting Wu^*^, Ling Chen, Zhang-bin Tan, Hui-jie Fan, Ling-peng Xie, Wen-tong Zhang, Hong-mei Chen, Jun Li

*** Correspondence:** Ying-chun Zhou, Email: zhychun@126.com.

Bin Liu, Email: xmhoolv@163.com.

# Supplementary Figures

##
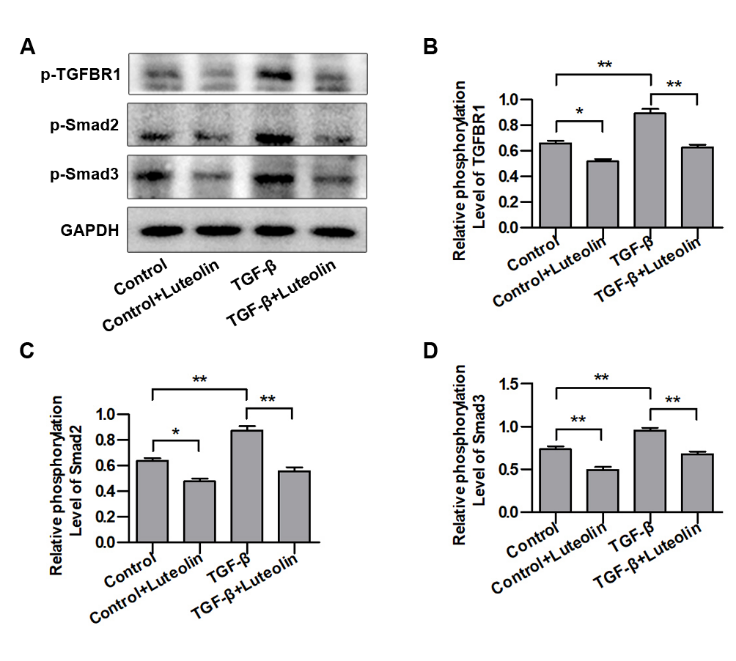


**Supplementary Figure 1.** **Luteolin** **suppresses TGF-β-induced TGFBR1/ Smads signaling activation.** (A) HASMC cells were incubated with luteolin (40 µM) for 1 h, and then co-treated with TGF-β for 15min. The expression levels of p-TGFBR1, TGFBR1, p-Smad2, Smad2, p-Smad3, and Smad3 were tested by western blotting. (B­–D) Relative phosphorylation levels of TGFBR1, Smad2, and Smad3 (n = 3). Data are presented as the mean ± SD. *P < 0.05, **P < 0.01.


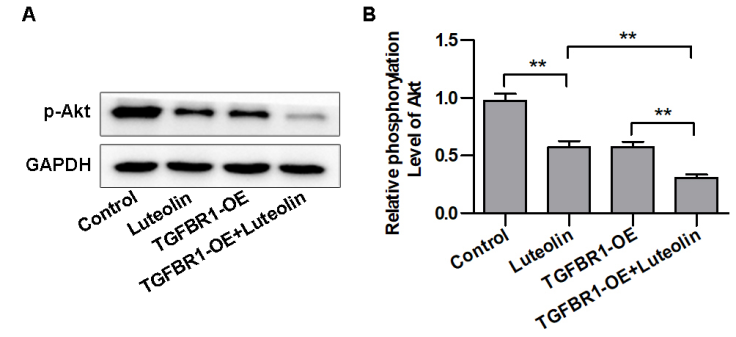


**Supplementary Figure 2. Overexpression of TGFBR1 did not rescue the Akt activation in luteolin-treated VSMC.** (A) HASMC cells was incubated with a TGFBR1 overexpression adenovirus vector and NC adenovirus for 48 h. Cells were then treated with luteolin (40 µM) for 1 h. The expression levels of p-Akt was tested by western blotting. (B) Relative phosphorylation levels of Akt (n = 3). Data are presented as the mean ± SD. *P < 0.05, **P < 0.01.
